# The Impact of Food and Nutrient-Based Standards on Primary School Children’s Lunch and Total Dietary Intake: A Natural Experimental Evaluation of Government Policy in England

**DOI:** 10.1371/journal.pone.0078298

**Published:** 2013-10-30

**Authors:** Suzanne Spence, Jennifer Delve, Elaine Stamp, John N. S. Matthews, Martin White, Ashley J. Adamson

**Affiliations:** 1 Institute of Health and Society, Human Nutrition Research Centre, Newcastle University, Newcastle upon Tyne, United Kingdom; 2 School of Mathematics and Statistics, Newcastle University, Newcastle upon Tyne, United Kingdom; 3 Institute of Health and Society, Newcastle University, Newcastle upon Tyne, United Kingdom; 4 Fuse, UKCRC Centre for Translational Research in Public Health, Newcastle upon Tyne, United Kingdom; University College Dublin, Ireland

## Abstract

In 2005, the nutritional content of children’s school lunches in England was widely criticised, leading to a major policy change in 2006. Food and nutrient-based standards were reintroduced requiring primary schools to comply by September 2008. We aimed to determine the effect of the policy on the nutritional content at lunchtime and in children’s total diet. We undertook a natural experimental evaluation, analysing data from cross-sectional surveys in 12 primary schools in North East England, pre and post policy. Dietary data were collected on four consecutive days from children aged 4–7 years (*n* = 385 in 2003–4; *n* = 632 in 2008–9). We used linear mixed effect models to analyse the effects of gender, year, and lunch type on children’s mean total daily intake. Both pre- and post-implementation, children who ate a school lunch consumed less sodium (mean change −128 mg, 95% CI: −183 to −73 mg) in their total diet than children eating home-packed lunches. Post-implementation, children eating school lunches consumed a lower % energy from fat (−1.8%, −2.8 to −0.9) and saturated fat (−1.0%; −1.6 to −0.5) than children eating packed lunches. Children eating school lunches post implementation consumed significantly more carbohydrate (16.4 g, 5.3 to 27.6), protein (3.6 g, 1.1 to 6.0), non-starch polysaccharides (1.5 g, 0.5 to 1.9), vitamin C (0.7 mg, 0.6 to 0.8), and folate (12.3 µg, 9.7 to 20.4) in their total diet than children eating packed lunches. Implementation of school food policy standards was associated with significant improvements in the nutritional content of school lunches; this was reflected in children’s total diet. School food- and nutrient-based standards can play an important role in promoting dietary health and may contribute to tackling childhood obesity. Similar policy measures should be considered for other environments influencing children’s diet.

## Introduction

The causes, complexities and adverse health effects of obesity are well documented [1]–[5]. Diet has played a significant role in contributing to childhood obesity levels in the United Kingdom [4]. The National Child Measurement Programme identified 23% of reception (4–5 year olds) and 33% of year 6 (10–11 year olds) children in England as overweight or obese in 2011 [6]. National Diet and Nutrition Surveys report children’s diets exceed recommended intakes of per cent energy from saturated fat and non-milk extrinsic sugars (NMES), and contain low levels of some micronutrients, such as iron [7], [8]. Central to improving children’s diets is the need to reduce intakes of fat, saturated fat, and NMES, while increasing nutrient density.

Although the food children consume at home is of great importance, up to a third of children’s daily energy and micronutrient intake is provided by school lunch [9]. Over the last four decades, policy changes have had a significant impact on the nutritional quality of school lunches in England. The 1980 Education Act removed nutritional standards, first introduced in 1941. Despite government introducing food-based standards for school lunches in 2001, [10] findings from a national survey of primary and secondary school lunches reported they contained too much fat and sugar, and lacked key micronutrients [9], [11]. In February 2005, TV chef Jamie Oliver’s media broadcast “Jamie’s School Dinners” attracted both public and Government attention and led to intensive lobbying by parents and pressure groups [12], [13]. In March 2005, a national School Meal Review Panel was established to advise on school food, [14] and in April of the same year the School Food Trust was established to “transform school food” [15]. A major policy change ensued in England, which received legislative support in 2006 [16]. New food- and nutrient-based standards were introduced and primary schools were expected to comply by September 2008 [17]. Food-based standards specify which foods can and cannot be served, and how often. Nutrient-based standards apply to the average nutritional content of school lunches over three-weeks, and specify minimum and maximum levels [18]. Both food- and nutrient-based standards focus on planned provision, not consumption.

To date, research has focused on changes to school and packed lunch [19]–[22]. There is a lack of research in the UK examining the wider effects of this important policy change, i.e. on the impact of school food standards on children’s total dietary intake. In this paper, we report a natural experimental evaluation [23] to assess whether the introduction of food- and nutrient-based standards in primary schools had an impact on children’s lunchtime dietary intake and their total diet.

## Methods

### Ethics Statement

Ethical approval was granted by Newcastle University ethics committee (reference 000011/2007). Parents provided written informed consent prior to children’s participation.

### Study Design, Setting and Participants

We undertook cross-sectional surveys during two academic years: 2003–4 (pre-) and 2008–9 (post-implementation) in 12 primary schools, North East England. The pre-implementation survey had been completed as part of a prior study [24]. The post-implementation survey used the same methods, which are described briefly here.

A letter with study details was posted to head teachers of the 16 primary schools that had participated in 2003–4. The results presented in this study are based on 12 schools for which comparable data were available from the two surveys. These schools had been identified to represent a comprehensive range of socio-economic circumstances, determined using the free school meal index at school level [25].

All children in Reception, Year 1 and Year 2 (aged 4–7 years) were eligible to participate. Each child received a letter with study details and a form requesting parental permission to participate in the study: consent forms were collected from schools by the study nutritionist.

### Data Collection

We used a prospective, 24-hour food diary method (the *Food Assessment in Schools Tool* (*FAST*)), validated to record young children’s dietary intake [24]. FAST assesses foods within six defined daily time slots, along with age- and sex-specific portion sizes, derived from the National Diet and Nutrition Surveys (NDNS) [7].

Four consecutive days of dietary consumption were assessed: three week days and one weekend day. Full written instructions on how to complete the diary were provided to parents. At each school, a team of trained observers and the study nutritionist recorded dietary intake, including, breakfast and afterschool clubs. The diary design enabled categorisation of foods into ‘school lunch’, ‘packed lunch’, and ‘food eaten at home’. All dietary coding for nutritional composition was based on McCance and Widdowson’s Integrated Composition of Food Dataset [26]. School recipes and menus were obtained to allow for coding of school food and assessing compliance with food- and nutrient-based standards.

All nutrients reported were checked for completeness in McCance and Widdowson’s Integrated Composition of Food Database [26]. To ensure consistency of dietary coding, all food codes, weights and food groups allocated were exported and interrogated, allowing identification and correction of inconsistencies.

### Main Outcome Measures

Main outcome measures were changes in mean daily intakes of macro- and micro-nutrients in school lunch, packed lunch and total diet. The values for vitamins A and C had skewed distributions and were log-transformed before analysis.

### Statistical Analysis

The sample size of the study was pragmatic and determined by the number of children studied in the earlier survey of the participating schools, and by the number of these schools prepared to participate in the more recent survey. Similar studies with smaller numbers of children aged 11–12 years have identified important and statistically significant changes in selected macro and micronutrients [27]–[29].

The first analysis assessed the direct effect of changes in school lunch standards, and considered only children who ate school lunches. The mean intake of macro- or micro-nutrients of each child from this source alone were compared between the 2003–4 and 2008–9 surveys. A more detailed analysis considered the intake of macro- and micro-nutrients from the total diet: this analysis explored the importance of year of the survey, whether the child ate a school or packed lunch, and the interaction between these factors. All analyses adjusted for the effect of gender and used a linear mixed effect model, with year (of survey), gender and packed/school lunch taken as fixed effects: potential correlation between responses within the same school and child were accommodated by fitting random effects for school and child. The models were fitted using *xtmixed* in Stata (version 10) and lme in R (version 2.14.0).

## Results

**Figure 1:**
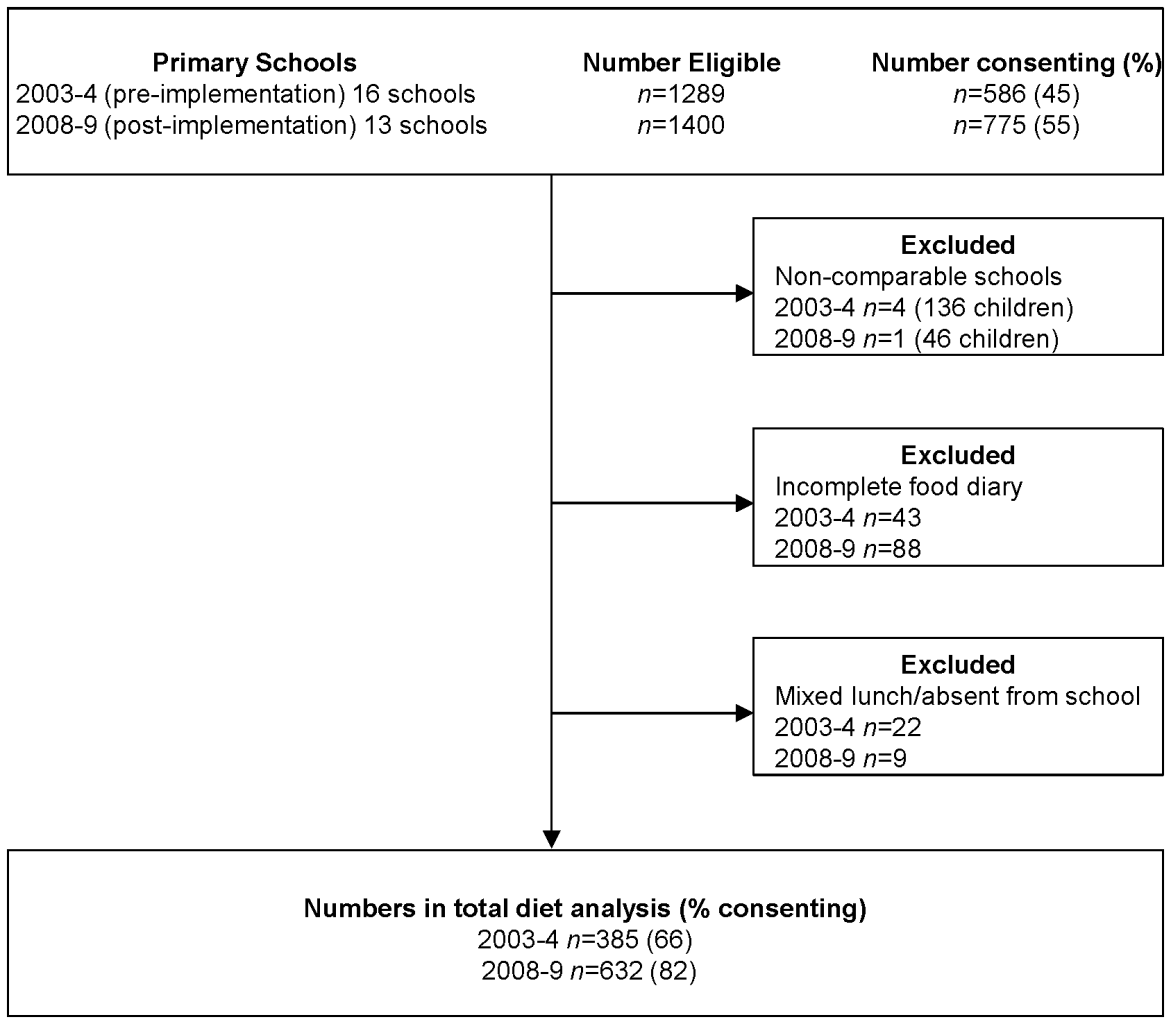
Flowchart detailing numbers (%) of children eligible, consenting, completing four-day food diaries and included in analysis.

Across all 12 schools, 586 (45% of those eligible) and 775 (55%) children consented in 2003–4, and 2008–9 respectively. Children eligible, consenting and completing, and reasons for exclusion are shown in Figure 1. The analyses included observed dietary intake from 407 children (boys *n* = 198; girls *n* = 209) in 2003–4, and 641 children (boys *n* = 322; girls *n* = 319) in 2008–9. In 2003–04, 233 children ate school lunch (boys *n* = 106; girls *n* = 127); in 2008–09, 323 children ate school lunch (boys *n* = 164; girls *n* = 159).

### Lunchtime Intake: Change in Mean Daily Nutrients from School Lunches between 2003–4 and 2008–9

Table 1 shows the change in children’s mean daily macro- and micro-nutrient intake from school lunches. Between 2003–4 to 2008–9 there was a statistically significant fall in children’s mean daily per cent energy intake of fat (mean change −11.2%, 95% confidence interval −12.1 to −10.4), saturated fat (−5.3%, −5.8 to −4.7), and NMES (−1.3%, −1.9 to −0.7), despite a small increase in mean energy intake (44 kcals, 26.6 to 62.0). Post-implementation children’s mean daily intake of sodium fell; intakes of calcium, vitamin C, iron, zinc, vitamin A, and folate intake all increased; these were also statistically significant (Table 1). In relation to the planned nutrient-based standards children’s mean intake from calcium, iron, zinc and vitamin A remain below the minimum standard.

**Table 1:** School lunch: change in primary school children’s mean daily intake of nutrients from 2003–4 to 2008–9 compared with planned nutrient-based standards.

Nutrient	Standard	Mean*		Mean difference†	95% CI	P-value‡
		2003–4	2008–9	2008–9–2003–4		
		*n* = 233	*n* = 323			
Energy (kcals)	target 530	450	494	44.0	26.6, 62.0	<0.001
% energy Fat	n/a	39.5	28.3	−11.2	−12.1, −10.4	<0.001
% energy Sat Fat	n/a	15.3	10.0	−5.3	−5.8, −4.7	<0.001
% energy NMES	n/a	9.4	8.1	−1.3	−1.9, −0.7	<0.001
Fat (g)	Max 20.6	19.9	15.6	−4.3	−5.1, −3.5	<0.001
Saturated Fat (g)	Max 6.5	7.7	5.5	−2.2	−2.5, −1.8	<0.001
Carbohydrate (g)	Min 70.6	57.1	71.4	14.3	11.6, 16.9	<0.001
Protein (g)	Min 7.5	14.3	19.2	4.9	4.2, 5.7	<0.001
NSP (g)	Min 4.2	2.9	4.7	1.8	1.6, 2.0	<0.001
NMES (g)	Max 15.5	11.4	10.6	−0.8	−1.6, 0.0	0.05
Sodium (mg)	Max 499	530	463	−67.0	−94.2, −39.8	<0.001
Calcium (mg)	Min 193	133	166	33.0	21.4, 43.6	<0.001
Vitamin C (mg)†	Min 10.5	11.8	46.0	3.9	3.5, 4.3	<0.001
Iron (mg)	Min 3	1.8	2.3	0.5	0.4, 0.6	<0.001
Zinc (mg)	Min 2.5	1.4	1.7	0.3	0.2, 0.4	<0.001
Vitamin A (µg)†	Min 175	69.2	84.5	1.2	1.0, 1.5	0.03
Folate (µg)	Min 53	45.7	59.1	13.4	10.2, 16.7	<0.001

* Mean adjusted for gender.

† Arithmetic means and differences are reported except for vitamins A and C (highly skewed) where geometric means and ratios are given.

‡ Confidence intervals and P-value derived from a linear mixed effects model with random term for schools.

### Total Dietary Intake: the Effect of Year and Lunch Type on Mean Daily Nutrient Intake

The results of the analysis of total diet are tabulated in Tables 2–4. Table 2 presents the results showing the effect of year, Table 3 the effect of lunch type, and Table 4 presents those variables for which there was a significant interaction between year and lunch type.

**Table 2:** Total diet: effect of year on children’s mean daily nutrient intake compared with Dietary Reference Values/Reference Nutrient Intakes*.

Nutrient	DRV/RNI	Mean†		Mean difference‡	95% CI	P-value
		2003–4	2008−9	2008−9–2003−4		
		*n* = 385	*n* = 632			
% energy NMES	11	18.0	15.6	−2.4	−3.0, −1.7	<0.001
Fat (g)	n/a	60.3	50.8	−9.5	−11.0, −8.0	0.001
Saturated Fat (g)	n/a	25.6	21.6	−4.0	−4.8, −3.3	<0.001
NMES (g)	60	77.5	60.6	−16.9	−20.3, −13.7	<0.001
Sodium (mg)	700	2000	1852	−148	−202, −93	<0.001
Vitamin A (µg)‡	400	210	224	1.08	1.00, 1.16	0.05
Calcium (mg)	450	677	669	−8.0	−33, 18	0.57
Iron (mg)	6.1	6.8	6.7	0.1	−0.2, 0.2	0.73
Zinc (mg)	6.5	4.9	4.9	0.0	−0.2, 0.1	0.77

* Dietary reference value/reference nutrient intake^35.^

† Mean adjusted for gender and lunch type.

‡ Geometric mean and ratio reported for vitamin A.

**Table 3:** Total diet: effect of lunch type on children’s mean daily nutrient intake compared with Dietary Reference Values/Reference Nutrient Intakes*.

Nutrient	DRV/RNI	Mean†		Mean difference‡	95% CI	P-value
		Packed Lunch	School Lunch	SL - PL§		
		*n* = 461	*n* = 556			
% energy NMES	11	17.9	15.3	−2.6	−3.2, −1.9	<0.001
Fat (g)	n/a	55.0	53.8	−1.2	−2.7, −0.4	0.13
Saturated Fat (g)	n/a	23.9	22.4	−1.5	−2.2, −0.8	<0.001
NMES (g)	60	72.6	62.3	−10.3	−13.6, −7.0	<0.001
Sodium (mg)	700	1978	1850	−128	−183, −73	<0.001
Vitamin A (µg)‡	400	206	230	1.12	1.04, 1.20	0.002
Calcium (mg)	450	688	659	−29	−54, −4	0.02
Iron (mg)	6.1	6.7	6.8	0.2	0.0, 0.4	0.12
Zinc (mg)	6.5	4.8	5.0	0.2	0.0, 0.3	0.02

* Dietary reference value/reference nutrient intake^35.^

† Mean adjusted for gender and year.

‡ Geometric mean and ratio reported for vitamin A.

§ SL (school lunch) PL (packed lunch).

**Table 4:** Total diet: effect of year and lunch type on children’s mean daily nutrient intake compared with Dietary Reference Values/Reference Nutrient Intakes*.

Nutrient	DRV/RNI*	Mean†				Mean‡		Difference between differences	95% CI	P-value
		2003–4		2008–9		2003–4	2008–9			
		*n* = 385		*n* = 632						
		SL^II^	PL^II^	SL	PL	SL-PL	SL-PL	[2008–9 SL-PL] – [2003–4 SL-PL]		
Energy (kcals)	§	1568.8	1625.3	1452.7	1423.6	−56.5	29.1	85.6	15.2, 156.1	0.02
% energy Fat	35	34.1	33.5	30.8	32.1	0.6	−1.2	−1.8	−2.8, −0.9	<0.001
% energy Sat Fat	11	14.3	14.5	12.8	14.0	−0.2	−1.2	−1.0	−1.6, −0.5	<0.001
Carbohydrate (g)	n/a	224.7	236.8	211.1	206.7	−12.1	4.3	16.4	5.3, 27.6	0.004
Protein (g)	19.7	47.2	47.1	50.3	46.6	0.1	3.7	3.6	1.1, 6.0	0.004
NSP (g)	n/a	8.7	8.5	10.5	8.8	0.2	1.7	1.5	0.5, 1.9	0.001
Vitamin C (mg)‡	30	58.1	67.8	89.0	72.6	1.2	0.8	0.70	0.60, 0.81	<0.001
Folate (µg)	100	163.3	160.0	171.7	155.9	3.5	15.8	12.3	9.7, 20.4	0.03

* Dietary reference value/reference nutrient intake^35.^

† Mean adjusted for gender.

‡ Geometric mean and ratio reported for vitamin C.

§ Boy (1715 kcals), Girl (1545 kcals).

II SL (Total intake of children having School Lunch), PL (Total intake of children having Packed Lunch).

In children’s total dietary intake between 2003–4 and 2008–9, there was a statistically significant reduction in mean daily intake of per cent energy from NMES (mean change −2.4%, 95% confidence interval −3.0 to −1.7), and in absolute intakes of fat (−9.5 g, −11.0 to −8.0), saturated fat (−4 g, −4.8 to −3.3), NMES (−16.9 g, −20.3 to −13.7), and sodium (−148 mg, −202 to −93). There was no evidence of a change in children’s mean daily intake of vitamin A, calcium, iron, or zinc (Table 2). In 2008–9 children’s mean daily intake of per cent energy from NMES and absolute amounts of sodium (mg) remain above the dietary reference value. Mean daily intakes of vitamin A (µg) and zinc (mg) remain below the dietary reference value.

Table 3 shows the effect of children’s lunch type (school or home-packed lunch) on their mean total dietary intake adjusted for year (pre- and post-implementation). Children who ate school lunch consumed a lower mean per cent energy from NMES (mean change −2.6%, 95% confidence interval, −3.2 to −1.9) and lower absolute intakes of saturated fat (−1.5 g, −0.8 to −2.2), NMES (−10.3 g, −13.6 to −7.0), and sodium (−128 mg, −183 to −73) than children eating a packed lunch. Mean daily intakes of vitamin A and zinc were higher in the total diets of children who ate a school lunch (Table 3). Although total fat intake was slightly lower and iron slightly higher in children who ate a school lunch, there was no statistically significant change. Children who ate a school lunch had a statistically significant lower intake of calcium (−29 mg, −54 to −4) in their mean total daily intake.

For a number of macro- and micro-nutrients examined, there was a significant interaction between year (pre- and post-implementation), and lunch type (school or home-packed lunch), and the consequent effect on total dietary intakes (Table 4). In 2003–4, children who ate a school lunch had a lower mean daily energy intake compared with children consuming a packed lunch (−57 kcals); by 2008–9, children who had a school lunch had a slightly higher mean daily energy intake, though this difference was very small (29 kcals). In 2003–4, children who ate a school lunch had a higher per cent energy from fat (0.6%); by 2008–9 children who ate a school lunch had a lower per cent energy from fat in their total diets than those who ate a packed lunch (−1.2%). Mean total daily per cent energy intake from saturated fat was lower in children who ate a school lunch in 2003–4 and remained lower in 2008–9 (Table 4). Carbohydrate and vitamin C intakes were lower in 2003–4 in those consuming school lunches; by 2008–9 children who ate a school lunch had a higher intake. In 2008–9 children who ate a school lunch had significantly higher mean daily total intakes of protein, NSP, and folate than children who ate packed lunches (Table 4). There was no statistically significant interaction between year and lunch type and children’s mean daily total intake of per cent energy from NMES and absolute amounts of fat, saturated fat, NMES, sodium, vitamin A, calcium, iron, or zinc.

## Discussion

### Summary of Main Findings

This natural experimental evaluation of the nutrient standards for primary schools in England identified important reductions in both per cent energy and absolute intakes of fat, saturated fat, and NMES in school and packed lunches post-implementation. While we observed a small increase in the energy content of a child’s average school lunch post-implementation, the average energy content provided by either a school or packed lunch was similar post-implementation (494 and 504 kcals respectively) and remained below the target stated in the requirements of 530 kcals/day. Post-implementation the average level of all micro-nutrients except calcium were higher in school lunches than packed lunches.

A number of these key changes in children’s mean daily intake from school lunch were reflected in children’s total diet. Post-implementation a child who ate a school lunch had a lower per cent energy derived from fat and saturated fat, but more carbohydrate, protein, NSP, vitamin C and folate in their total diet than children who ate a packed lunch. Findings show that children mean daily intake of % energy from saturated fat and NMES, and absolute amounts of sodium remain above Dietary Reference Values. Children’s mean daily intake of Vitamin A and Zinc continue to remain below the Reference Nutrient Intake.

### Strengths and Weaknesses of the Study

This natural experimental evaluation was dependent on repeated cross-sectional surveys, and as such, we were limited in the extent to which changes in nutrient intakes could be attributed to the implementation of the school food policy. Externally imposed time constraints for the implementation of the new standards precluded a stronger, prospective study design. Nevertheless, the study offers a unique evaluation of national policy, enabled by the availability of pre-implementation data, collected for an earlier study [24]. To avoid introducing measurement bias, the same methods were employed post-implementation. The study was restricted to a sample of primary schools in one city in North East England, which potentially limits generalisability.

Food and nutrient-based standards for primary schools are based on the average school lunch over a three-week menu cycle. Some foods on which standards are based, such as oily fish, only have to be served once in this three week cycle. A potential limitation to assessing the impact of food and nutrient-based standards on children’s total diet is that our data collection did not cover a full three week cycle in primary schools. However, there is a selection of food items available at school lunch each day. Children make choices both at the counter and, once seated, they may or may not choose to consume particular food items served. Our findings are based on children’s actual food consumption.

### Relationship to Previous Work

This study has shown changes in the nutrient content of both school and packed lunches, but also provides evidence of a widening gap between school and packed lunches. The finding that packed lunches contained more fat, saturated fat, sodium and NMES than school lunch confirms the findings of previous studies [20],[30]–[33]. This study, along with others, [19], [32], [33] provides some evidence of the potential advantages of planned, nutrient-based lunch provision compared with home-prepared packed lunches. Our findings on total diet are similar to those of the NDNS [34] and show some improvements in children’s nutrient intake over recent years. This study provides evidence that at least part of this improvement is associated with the change in school food policy. Although this study has not reported on children’s weight gain following the implementation of the standards, a recent study in the US examined the impact of stricter nutritional standards and student weight gain [35]. Their findings show that, in states with stringent regulation of school food, children eating school lunches improved their weight status. This adds further support for regulation of foods offered at school lunch and the potential impact of such legislation on child health.

### Future Research

Both the Healthy Lives, Healthy People (2010) [36] and Foresight [4] reports have highlighted the issue of social inequalities in children’s diets. Schools offer a unique opportunity to influence the food choices of all children with the potential to reduce inequalities [37]. Further research is needed to assess whether the introduction of new school food- and nutrient-based standards has had a comparable effect on children’s total diet across the socio-economic spectrum.

### Conclusions and Implications

Although our findings show reductions in children’s average daily intake of per cent energy from saturated fat and NMES, and absolute intakes of NMES and sodium, intakes remain above the Dietary Reference Values [38]. These remain key areas for public health action, necessitating a focus on children’s food choice at school and beyond. At school, more encouragement and supervision of children at lunchtime with selection of foods, more time to eat, and more child friendly dining environments have been advocated [39]. Following implementation of the nutritional standards, school lunches appeared to have a positive impact on children’s total diet, but this can only be realised fully in children who eat school lunch. School lunch competes against packed lunch where children bring their choice of foods. Although it was observed some schools do impose rules (e.g. no sweets, chocolate, or crisps), there are more often no regulations as to what can and cannot be brought from home in a packed lunch.

It has been advocated that to address the complexity of obesity there is a need for political will [1], [5], [40], [41]. In 2011, Swinburn et al [39] commented that to enable ‘healthy choices’, policy interventions are required at the environment level. After a highly publicised campaign on the state of school lunches, government provided legislative and financial support for this change in policy, thereby creating an environment to enable healthier food choices in schools. Within the limitations of the natural experimental design, we found that children’s total diet has improved since the reintroduction of food- and nutrient-based standards. Our findings of a positive effect on both lunchtime and total diet intake provide evidence to support this level of intervention in primary schools. Similar policy approaches should be considered for other schools and academies, and other environments influencing children’s diet outside school. Prospective evaluation of public health policy interventions would add considerably to the evidence base.
